# Using single-cell sequencing technology to detect circulating tumor cells in solid tumors

**DOI:** 10.1186/s12943-021-01392-w

**Published:** 2021-08-19

**Authors:** Jiasheng Xu, Kaili Liao, Xi Yang, Chengfeng Wu, Wei Wu, Shuwen Han

**Affiliations:** 1 Department of Oncology, Huzhou Central Hospital, Affiliated Central Hospital Huzhou University, No.1558, Sanhuan North Road, Wuxing District Zhejiang Province, Huzhou, China; 2 Department of Vascular Surgery, the Second Affiliated Hospital of Nanchang University, No. 1 Minde Road, Nanchang, 330006 Jiangxi China; 3 Department of Clinical Laboratory, the Second Affiliated Hospital of Nanchang University, No. 1 Minde Road, Nanchang, 330006 Jiangxi China; 4 Department of Gastroenterology, Huzhou Central Hospital, Affiliated Central Hospital Huzhou University, No.1558, Sanhuan North Road, Wuxing District Zhejiang Province, 313000 Huzhou, China

**Keywords:** Circulating tumor cells, Single-cell sequencing, Tumor heterogeneity, Research progress

## Abstract

Circulating tumor cells are tumor cells with high vitality and high metastatic potential that invade and shed into the peripheral blood from primary solid tumors or metastatic foci. Due to the heterogeneity of tumors, it is difficult for high-throughput sequencing analysis of tumor tissues to find the genomic characteristics of low-abundance tumor stem cells. Single-cell sequencing of circulating tumor cells avoids interference from tumor heterogeneity by comparing the differences between single-cell genomes, transcriptomes, and epigenetic groups among circulating tumor cells, primary and metastatic tumors, and metastatic lymph nodes in patients' peripheral blood, providing a new perspective for understanding the biological process of tumors. This article describes the identification, biological characteristics, and single-cell genome-wide variation in circulating tumor cells and summarizes the application of single-cell sequencing technology to tumor typing, metastasis analysis, progression detection, and adjuvant therapy.

## Introduction

Circulating tumor cells (CTCs) are tumor cells with high vitality and high metastatic potential that originate from primary or metastatic tumors of epithelial origin and shed into the blood circulation system. CTCs are one of the important components of liquid biopsy, and provide a window to monitor tumor progression in real time [1–3]. Generally, high-throughput sequencing analysis of tumor tissue is based on the analysis of a mixed sample of millions of cells, which reflects the overall genomic characteristics of the cell but ignores the heterogeneity of the tumor cell, resulting in the dilution of genetic material from CTCs and cancer stem cells (CSCs) and other low-abundance but functionally important cells. However, the emergence of single-cell sequencing technology has solved this problem well [4–6]. Single-cell sequencing of CTCs can be used to compare the differences between single-cell genomes, transcriptomes and epigenetic groups in peripheral blood CTCs, tumor primary and metastatic foci, and metastatic lymph nodes, reducing interference from tumor heterogeneity. Single-cell sequencing provides a new perspective for understanding the biological process of tumor occurrence and development [7–9] and has been used in breast cancer, colorectal cancer, malignant melanoma, lung cancer and prostate cancer and other tumor research [10–14]. This article reviews the progression of research into the analysis of the genomic variation in CTCs in the peripheral blood of solid tumors using single-cell sequencing technology.

## Tumor heterogeneity and single-cell sequencing analysis

Tumor heterogeneity refers the genomic, proteomic, and gene expression differences in daughter cells that occur during the growth process of the tumor after multiple divisions and proliferation; these differences can induce differences in phenotypes and features, such as growth rate, invasion ability, drug sensitivity and prognosis. Heterogeneity is a characteristic of malignant tumors. Tumor heterogeneity can allow tumor cells to adapt to changes in the tumor microenvironment and promote tumor resistance and progression.

**Fig. 1:**
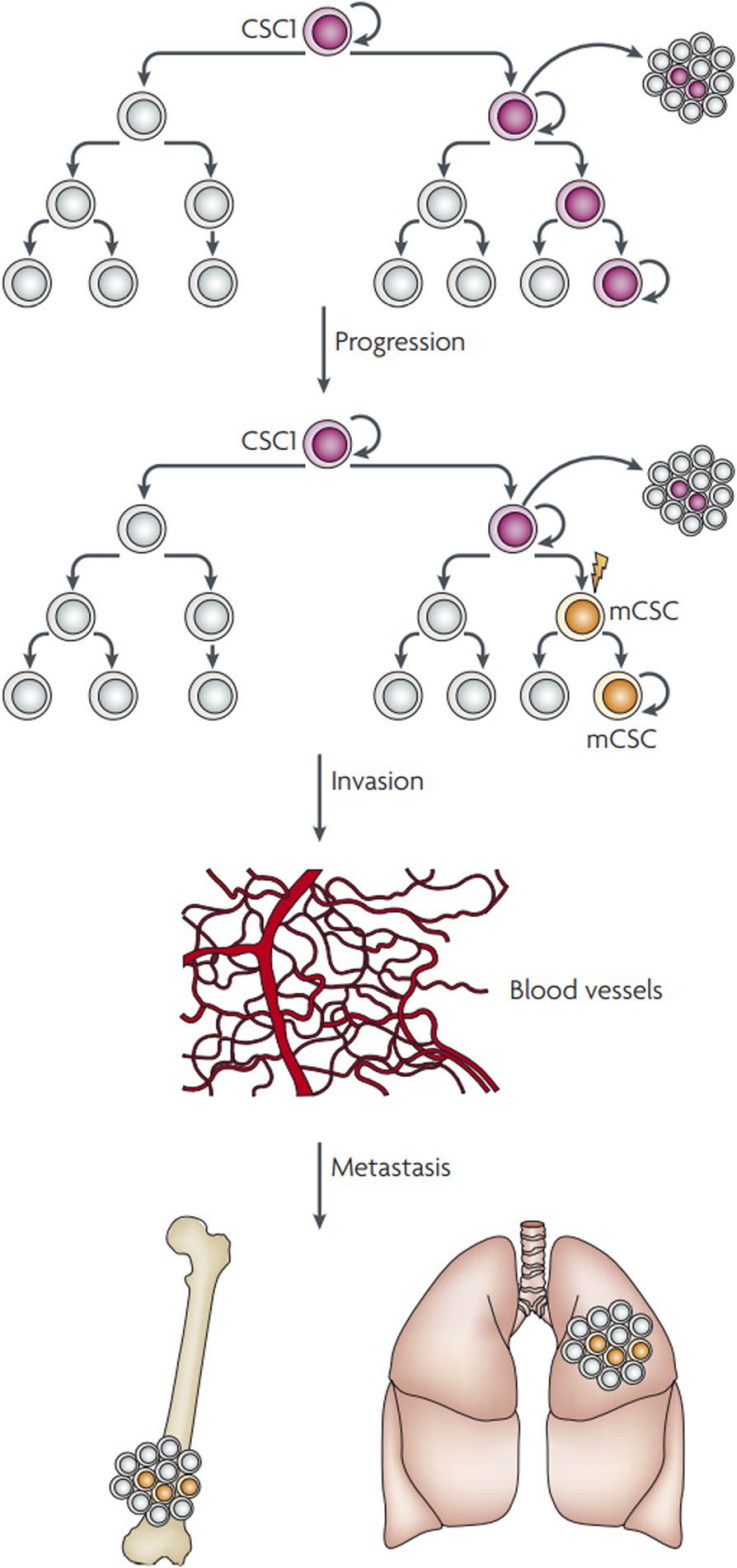
Evolution of a metastatic cancer stem cell (CSC). As a tumour progresses, genetic and epigenetic mechanisms may result in the emergence of a selfrenewing metastatic CSC (mCSC) that expresses different cell surface markers from the CSC that is driving tumorigenesis. This mCSC, through a series of invasive processes that characterize metastasis, enters the blood stream and seeds a secondary tumour in a distinct organ [18]. The copyright of this image belongs to Reference [18].

In general, tumor heterogeneity consists of two types: intratumor heterogeneity, composed of differences in genes and phenotypes between different cells within a tumor, and intertumor heterogeneity composed of differences in genes and phenotypes between cells in different tumors. Intratumor heterogeneity refers to the presence of different cancer cell subpopulations in the same tumor. The genomics of different tumor cell subsets (such as genome, somatic mutation and epigenetic modification, etc.) and tumor biology (transcriptome, proteome, metabolome, etc.) are significantly different [15]. Not only tumor cells but also the tumor microenvironment (such as lymphocyte infiltration and MHC molecular types, etc.) and the interaction between tumor cells and the tumor microenvironment are different [16]. Heterogeneity within the tumor allows tumor cells to have both temporal heterogeneity (the primary tumor is different from the secondary tumor) and spatial heterogeneity (the same tumor differs in different regions). It is currently believed that heterogeneity in tumors is related to the randomness of gene mutations and the heterogeneity of environmental factors and their effects [17]. The clonal evolution theory speculates that normal cells in an organism become cancer cells through various genetic mutations, in which clones multiply and form identical copies, and each copy has the same oncogenic ability (Fig. 1) [18, 19]. Heterogeneity between tumors refers to the difference between tumors of the same origin in different patients. These tumor subgroups have special molecular markers and different biological behaviors, thus causing different effects on clinical prognosis [20]. Heterogeneity between tumors stems from different tumor phenotypes caused by differences in the responses of tumor cells to genomic and epigenetic modifications and different tumor cell subsets originating from different tumor stratifications [21]. In addition, tumor interstitial heterogeneity is also related to the abnormal regulation of cells and extracellular matrix in the tumor microenvironment. For example, the tumor interstitium contains different tumor-associated fibroblasts, macrophages, and tumor-infiltrating lymphocytes. All these factors play an important role in the malignant transformation of tumors [22].

Studies have revealed that there are multiple levels of heterogeneity in the primary tumor, metastatic lymph nodes, metastases, and different metastases, including genomic variation, RNA transcription, and protein expression profiles. In 2012, Vermaat et al. [23] used a customized "Cancer Mini-Genome" chip to sequence the exome of 1264 genes in tumor-related signaling pathways in primary colorectal cancer and liver metastasis samples. The results showed that the number of variants affecting protein function in the primary tumor was far greater than that in liver metastasis, revealing that the genomic differences between the primary foci of colorectal cancers and their liver metastases were substantial. In addition, studies have also shown that KRAS, BRAF, and PIK3CA mutations and PTEN expression in primary and metastatic foci of colorectal cancer, as well as EGFR pathway status, were significantly different [24, 25].

Colombino and others [26] compared the mutations in the primary and metastatic lymph nodes of malignant melanoma patients, as well as the BRAF, NRAS and p16CDKN2A mutations in brain and skin metastases, and found that the metastatic lymph nodes were more similar to the primary lymph nodes than to the brain and skin metastases. Similar results have been shown in the study of breast cancer and lung adenocarcinoma. The expression of HER2 in breast cancer and the mutation rate of EGFR in lung adenocarcinoma were significantly among between primary tumors, metastatic foci, and different metastases. [27, 28].

The heterogeneity of tumors has been clarified by high-throughput sequencing analyses of mixed samples of tumor tissue, which mainly reflects the overall genomic characteristics of tumor cells. The heterogeneity of tumor cells causes a low abundance of CTCs and cancer stem cells [29]. However, the genomic characteristics of functionally important cells are unclear. Single-cell sequencing analysis of tumor tissues and CTCs is necessary to understand the biological progression of tumors.

## Circulating tumor cells

CTCs are a group of tumor cells with high activity and high metastatic potential in the peripheral blood of solid tumor patients. CTCs are one of the important tumor markers in tumor liquid biopsy. Both the number of CTCs and their phenotypes are related to the progression of the primary tumor. Observing and analyzing the number and phenotypes of CTCs can indirectly reveal the nature of tumor lesions. The view that CTCs can be used to monitor tumor progression through peripheral blood analysis has been widely recognized [1–3]. Anatomists and pathologists have observed the presence of CTCs in the peripheral blood of patients with solid tumors.

Due to the low abundance of CTCs in peripheral blood relative to the number of blood cells, it is very difficult to distinguish them from other blood cells. With the progression of biological technologies, especially the developments of new nanomaterials and microfluidics, in 2004, the US Food and Drug Administration (FDA) approved a new technology [30] for detecting peripheral blood CTCs in metastatic breast cancer patients.

CTCs have been detected in the peripheral blood of patients with breast cancer, prostate cancer, liver cancer, lung cancer, ovarian cancer, esophageal cancer, pancreatic cancer, cervical cancer, colorectal cancer, head and neck cancer and gastric cancer.

### Enrichment and identification of circulating tumor cells

Separation of rare cells requires enrichment and capture of CTCs. CTC enrichment technology mainly includes enrichment based on cell surface markers and enrichment technology based on microfluidic chips. Enrichment based on cell surface markers mainly includes positive selection and negative selection, namely, anti-epithelial cell adhesion molecule (EpCAM) and keratin (cytokeratin, CK), and other antibodies capture and enrich epithelial-derived tumor cells and/or use leukocyte-derived antibodies to remove leukocytes. For example, the FDA approved the Cell Search enrichment method for CTC detection in breast cancer and prostate cancer patients using EpCAM and CD45 antibodies to capture EpCAM + cells from the blood. After removing the CD45 + cells and analyzing the isolated CTC count, CD45 + negative selection was also performed [1, 2, 31]. Another CTC capture technology uses a microfluidic chip. According to the biological and physical characteristics of CTCs, the peripheral blood mononuclear cells (PBMCs) of tumor patients were isolated and passed through a microfluidic chip coated with EpCAM antibody under stable and slow laminar flow control, and EpCAM + cells were captured by EpCAM antibody and bound to the bottom of the chip, while the remaining lymphocytes flowed out with the liquid, as in the CTCs-i Chip method [32, 33]. CTC identification is mainly performed by immunofluorescence, fluorescence in situ hybridization (FISH), and RT-PCR analysis. The isolated peripheral blood nucleated cells identified in previous studies showed features such as EpCAM + , CK + , and CD45- phenotypes by immunofluorescence staining, chromosomal aneuploidy changes, and specific tumor-related gene mutations. These cells were identified as CTCs derived from epithelial cells [2, 34, 35].

In addition, Swennenhuis et al. [34] developed a self-seeding microwell chip for the isolation and interrogation of single cells, which contained 6400 microwells, each microwell with a single 5 μM pore in the bottom. The cell suspension enters the microwell and drags a cell onto the pore. After identification by fluorescence microscopy, the cells of interest are isolated from the microwell by punching the bottom together with the cell. The overall single-cell recovery rate by seeding followed by isolation of the single cell is > 70% with a specificity of 100%, as confirmed by the genetic make-up of the isolated cells. Stevens et al. [35] developed Puncher technology for the isolation of single cells, which combines a silicon chip with microwells, fluorescence imaging, and a punching method to isolate and transfer single cells to standard reaction tubes. They used Rosettesep and Parsortix as pre-enrichment methods that are compatible with Puncher technology, which leads to high recovery rates when isolating a single CTC accurately from a small quantity of enriched samples. In contrast to other single-cell separation techniques, Puncher technology can be applied to isolate very low concentrations of single cells from liquid biopsies and, more generally, from cell suspensions.

### The main biological characteristics of circulating tumor cells

**Fig. 2:**
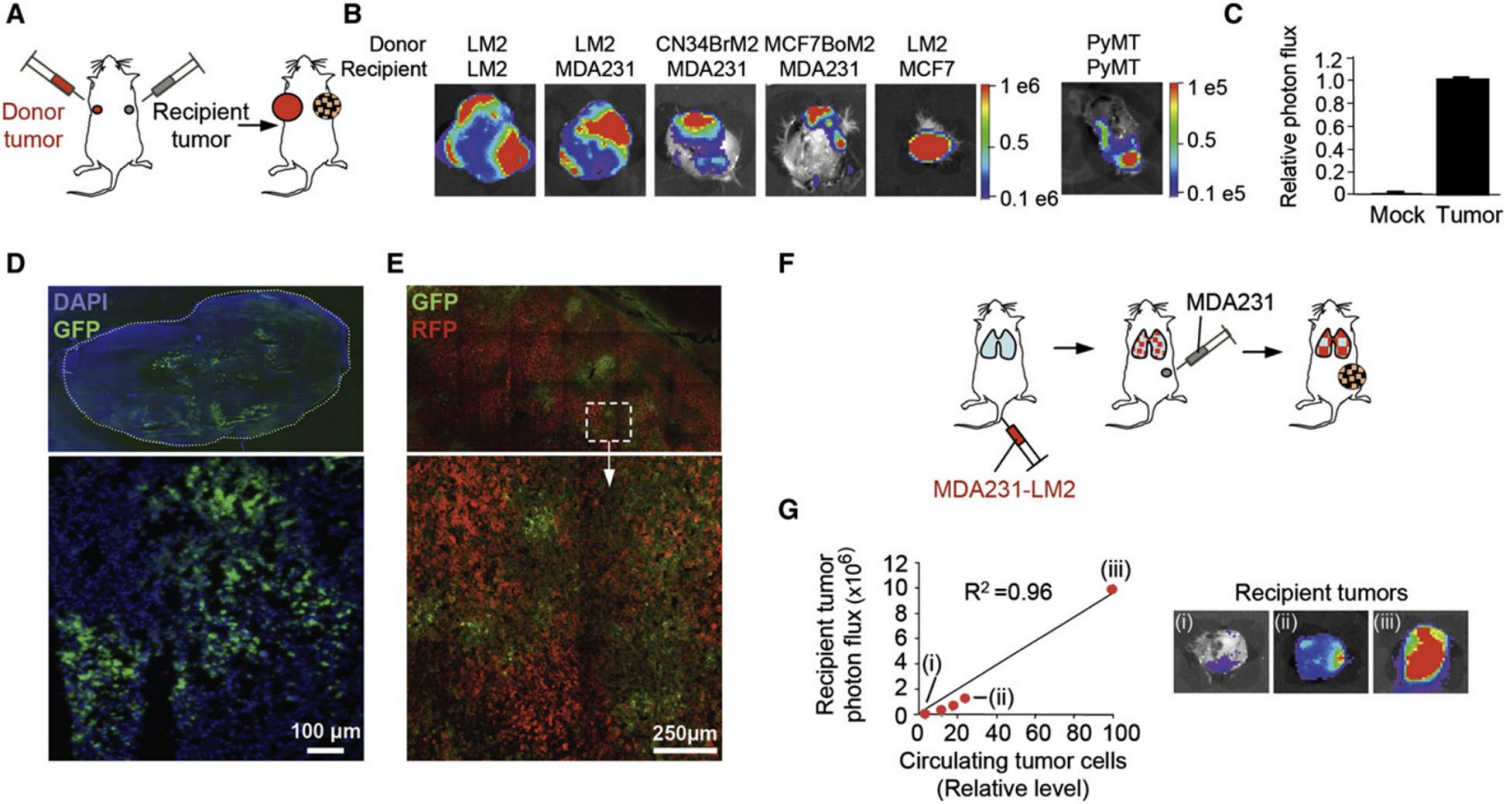
Seeding of established tumors by CTCs. **A** A diagram of contralateral-seeding experiment. Unlabeled and GFP/luciferase-expressing breast cancer cells were injected into contralateral No. 2 mammary glands as a “recipient tumor” and a “donor tumor”, respectively. **B** BLI of recipient tumors extracted from mice bearing the indicated GFP/luciferaseexpressing donor tumors. Color-range bars: photon flux. LM2: a lung metastatic derivative of MDA231. MCF7-BoM2: a bone-metastatic derivative of MCF7, CN34-BrM2: a brainmetastatic derivative of pleural effusion CN34, PyMT: cells derived from mammary tumors developed in MMTV-PyMT transgenic mice. **C** BLI of tumor-free and tumor-bearing mammary glands from mice bearing GFP/luciferaseexpressing donor tumors. n = 9–18. **D** Frozen sections of seeded MDA231-LM2 tumors were visualized by fluorescence microscopy. An entire tumor section and a higher-magnification image (× 10) of a selected field are shown. **E** A contralateral-seeding experiment was performed with RFP- and GFP-expressing. MDA231-LM2 cells. Frozen sections from RFP-labeled tumors were visualized under confocal microscopy at × 20. **F**A diagram to test mammary tumor seeding from lung metastases. GFP/luciferaseexpressing MDA231-LM2 cells were injected intravenously. Once lung metastases were established, unlabeled MDA231 cells were injected into a mammary gland No. 2. **G** Left: burden of CTCs derived from lung metastases in mice described in panel **F**. Relative levels of CTC were plotted against the luminescent signals of recipient tumors. Right: BLI of three representative recipient tumors (i, ii and iii) identified in the graph [36].The copyright of this image belongs to Reference [36]

Peripheral blood CTCs are also heterogeneous tumor cells. The surface markers of CTCs from different tumors vary, and the cells differ in size and can appear as single cells or cluster-like. Almost all of the invasive and metastatic CTC subpopulations survive in the circulation, lodge into distant sites and support the formation of a metastatic niche. Additionally, relatively benign CTCs have the advantage of increased replication rates compared with those of more aggressive but more vulnerable CTCs. Studies have shown that the appearance of CTCs in the peripheral blood is an early event in the formation of solid tumors of epithelial origin [30]; CTCs in the peripheral blood can simultaneously or bidirectionally stimulate the growth of tumor cells in primary and metastatic tumors (Fig. 2) [36]. CTCs appear cluster-like, which is the hallmark of tumor stem cells, and the presence of CTC clusters in the peripheral blood of tumor patients suggests tumor progression [37]. The clinical significance of detecting CTCs is mainly as follows. (1) CTCs are an important supplement for clinical tumor TNM staging. There are many deficiencies in TNM staging based on anatomical characteristics. In 2007, CTCs were recommended as a tumor marker by the American Society of Clinical Oncology (ASCO) as an important supplement to the TNM staging of tumors. Additionally, the 8th edition of the American Joint Committee on Cancer (AJCC) breast cancer staging manual released in 2017 affirmed the significance of CTCs. The peripheral blood CTC counts of clinically advanced breast cancer patients were ≥ 5 per 7.5 mL, and clinical early breast cancer patient peripheral blood CTC counts ≥ 1 per 7.5 mL indicated a poor prognosis. The level of evidence is grade II [1]. (2) Efficacy monitoring and prognosis judgment is another significant factor. CTCs can directly indicate the response of cancer patients to treatment. The continuous increase in CTC number indicates that the tumor responds poorly to treatment. The occurrence of CTCs in the peripheral blood of patients with malignant tumors indicates a poor prognosis. The occurrence or increase in CTCs after treatment is associated with tumor recurrence. (3) CTCs can be used to guide individualized treatment of tumors. The biological characteristics of metastatic foci are different from those of primary foci, the sensitivity of drugs can be detected by CTC ex vivo culture, and individualized treatment plans can be formulated according to the biological characteristics of the foci [38]. (4) Early warning of metastasis and recurrence can be provided by CTCs. Tumor metastasis is the main cause of death among cancer patients. The current methods for detecting tumor metastasis are mainly based on imaging. Even with high-resolution imaging methods, it is difficult to detect early tumor metastasis events at the cellular level. Through high-resolution imaging examination combined with dynamic monitoring of the number and nature of CTCs, potential metastasis clues in tumor lesions can be identified, which provides the possibility for early targeted treatment.

## Single cell sorting and sequencing analysis

### Single-cell sorting

Since peripheral blood CTCs are rare cells, some traditional single-cell sorting methods, such as fluorescence-activated cell sorting (FACS), are not suitable for CTC single-cell sorting. The methods used for CTC single-cell sorting mainly include the micromanipulation sorting method, microfluidic technology sorting method, DEPArray and Cell Celector sorting system (Table 1).

**Table 1:** Comparison of CTC single cell sorting methods

Sorting method	Sorting objects	time consuming	Advantage	shortcoming	Reference
Microoperation separation method	Less tissue and cell suspension	long	High accuracy and low cost	Time consuming, low flux, easy to cause mechanical damage to the target cells	[33, 34]
Microfluidic separation	Cell suspension with numerous cells	short	High flux, small reaction volume, less space and less pollution	High cost and high cell loss rate	[36, 39]
DEPArray sorting system	CTO solution	long	Visualization and semi automation	The sample size is small and can not be directly derived from it	[7, 40, 41]
				Separation and enrichment of CTC in peripheral blood	
CellCelector sorting system	CTC suspension	short	Keeping cell activity, high accuracy and less time consuming	High cost and high instrument dependence	[42]

Micropipette isolation (micropipette isolation) involves a high-power microscope that uses micromechanical manipulators or visual tweezers to complete single-cell sorting [40]. The advantage is that it can effectively control the selection, transfer, and release of target cells to ensure that the accuracy of cell selection at a low cost, but this method takes a long time with low flux, and it easily causes mechanical damage to the target cells. This method is suitable for the separation of target cells present at low numbers in the overall cell population and can be used for enrichment by Cell Search or Mag Sweeper technology for the sorting of nucleated cells [6, 41, 43]. The microfluidics sorting method (microfluidics) can be coupled with downstream genome amplification technology to complete single-cell sorting, lysis, and amplification in one step, such as Fluidigm's C1 single-cell amplifier with high throughput (each chip can be completed 96 single-cell sorting), small reaction volume (can increase amplification efficiency and reduce reagent consumption), less contamination and little impact on sequencing. The disadvantages are a low capture rate for viscous and nonspherical cells and a high chip cost [39, 42].

The DEPArray sorting system (Di-Electro-Phoretic Array system) refers to a semiautomatic sorting system that separates rare cells from a mixed cell population [44]. Visualizing the cells to be sorted by fluorescent labeling, single cells are captured by the "electronic cage" formed by the microelectrodes on the chip, and then the microelectrodes are turned on or off to move the sorted and captured target cells to a suitable position on the chip and place them in suitable media for subsequent sequencing analysis [45]. The disadvantage is that it takes a long time, and the sample volume is small (only 14 μL). Peripheral blood samples need to be divided and enriched in CTCs before they can be sorted by this system. This approach has been used at the single-cell level to study CTCs in breast cancer and colorectal cancer [7, 46, 47]. The Cell Celector sorting system is an automatic sorting system that separates rare cells from mixed cell populations. It automatically retrieves single cells and cell clones through a multifunctional robot system to achieve single-cell sorting and directly separates the target cells or clones mechanically without affecting cell viability, allowing for real-time, highly accurate observation of cell images for cell sorting; however, this method is time-consuming [48].

### Single cell whole genome amplification

The DNA content of a single cell is only 6 ~ 7 pg, which does not meet the level of DNA content required for whole-genome sequencing. High-fidelity, high-efficiency and unbiased genome amplification are required for whole-genome sequencing of a single cell. The development of whole-genome amplification (WGA) has promoted the progression of single-cell genome sequencing technology [49].

Based on the PCR amplification method, that is, the PCR amplification method that improves the specific primers or random primers of traditional PCR, such as primer-adapter PCR (LA-PCR), primer extension preamplification PCR, PEP-PCR and degenerate oligonucleotide primer PCR (degenerate oligonucleotides deprimed PCR, DOP-PCR), solves the problems of different primer annealing kinetics, low fidelity of the enzyme and exponential amplification, but insufficient coverage and unevenness of amplification products, amplification deviations, and allele deletions may cause single-nucleotide variation (SNV) and cause false positives [50, 51]. In addition, according to CTC enrichment techniques, the prevalence and metastatic potential of CTC subpopulations may differ, leading to different conclusions.

Multiple displacement amplification (MDA) uses random hexamers as primers to continuously synthesize φ29 DNA polymerase with strong synthesis ability, high fidelity, and strong strand displacement activity and completes the amplification at 30 °C [52, 53]. Under isothermal conditions, random primers with exonuclease activity are combined with the template. During amplification, φ29 DNA polymerase can replace the complementary strand of the template, and the substituted single-stranded DNA is further amplified as a new template, showing a branched structure. Exponential amplification is completed, and amplicon fragments of 5–10 kb are formed. MDA is a commonly used single-cell whole-genome amplification method with high coverage and uniformity, good accuracy, and long amplicons, but there are high allele deletion rates, exponential amplification-caused sequence-dependent deviations, and the approach neglects differences between cells. The lack of heterogeneity detection in this approach means that it is not suitable for detecting copy-number variation (CNV) [5, 54]. Many amplification methods are further improved on the basis of MDA, such as the microwell displacement amplification system (microwell displacement amplification system, MIDAS), through microfluidic technology to reduce the reaction volume, thereby reducing the deviation caused by the amplification, which can be reduced to a nanoliter volume. Thousands of single-cell genomes in microwells are amplified simultaneously, thereby increasing the uniformity of amplification reactions [55]. Emulsion whole-genome amplification (e WGA) disperses genomic DNA fragments into skin-emulsified emulsion droplets. Over time, each reaction system reaches saturation, reducing the dependence on amplification due to sequence differences. Compared with classic MDA, this method guarantees high coverage and improves accuracy and resolution [56]. Picher et al. [57] introduced the DNA primer enzyme Thermus thermophilus Prim Pol (Tth Prim Pol) into the MDA system to form a replication initiation polymer, which can simultaneously perform DNA chain initiation and extension functions. This WGA method was named True Prime. Compared with the original MDA method, this method increases the number of amplified products, and the resulting product fragments are longer and more sensitive to a small volume of initial DNA template. This method provides better coverage and uniformity, improved reproducibility, and can be used for CNV analysis. Additionally, the number of SNVs caused by allele dropout (ADO) decreased, and the false positive rate of SNV detection decreased. Chimeras are formed during the chain replacement process in the MDA reaction, but this method still relies on φ29 chain replacement activity, so there is no improvement in chimera formation levels [57].

Multiple annealing and looping-based amplification cycles (MALBAC) is a linear amplification method. After extension to form a semiamplicon, the temperature is raised to cause the product to fall off the template, and the temperature is reduced to form a hairpin structure to prevent further amplification, thereby ensuring that only amplification of the original template is performed, and the entire reaction cycle occurs 8 to 12 times, resulting in microgram-level genomic samples [58]. As this method involves linear amplification and high uniformity without amplification deviation, it is more suitable for the detection of CNVs. However, due to the low fidelity of the Taq DNA polymerase used, the false positive rate of SNV detection by this method is higher (approximately 40 times higher than that of MDA) [54].

The linear amplification via the transposon insertion (LIANTI) system introduced a specially designed Tn5 transposon that contains the T7 promoter that can be randomly combined with the genome; then, in vitro, linear amplification is reversed. Thousands of RNA copies are subsequently reversed and synthesized by the second strand to form the LIANTI amplicon for library construction. Compared with other amplification methods, the coverage and uniformity of this method are improved, and the allele deletion rate and false positive rate are reduced [59].

The DNA content of a single cell is at the pg level, so special attention should be paid to the prevention of contamination from the environment and the operator to reduce nonspecific amplification during amplification, and operation and control of contamination should be performed under sterile, controllable air pressure conditions.

### Single cell whole genome sequencing

**Fig. 3:**
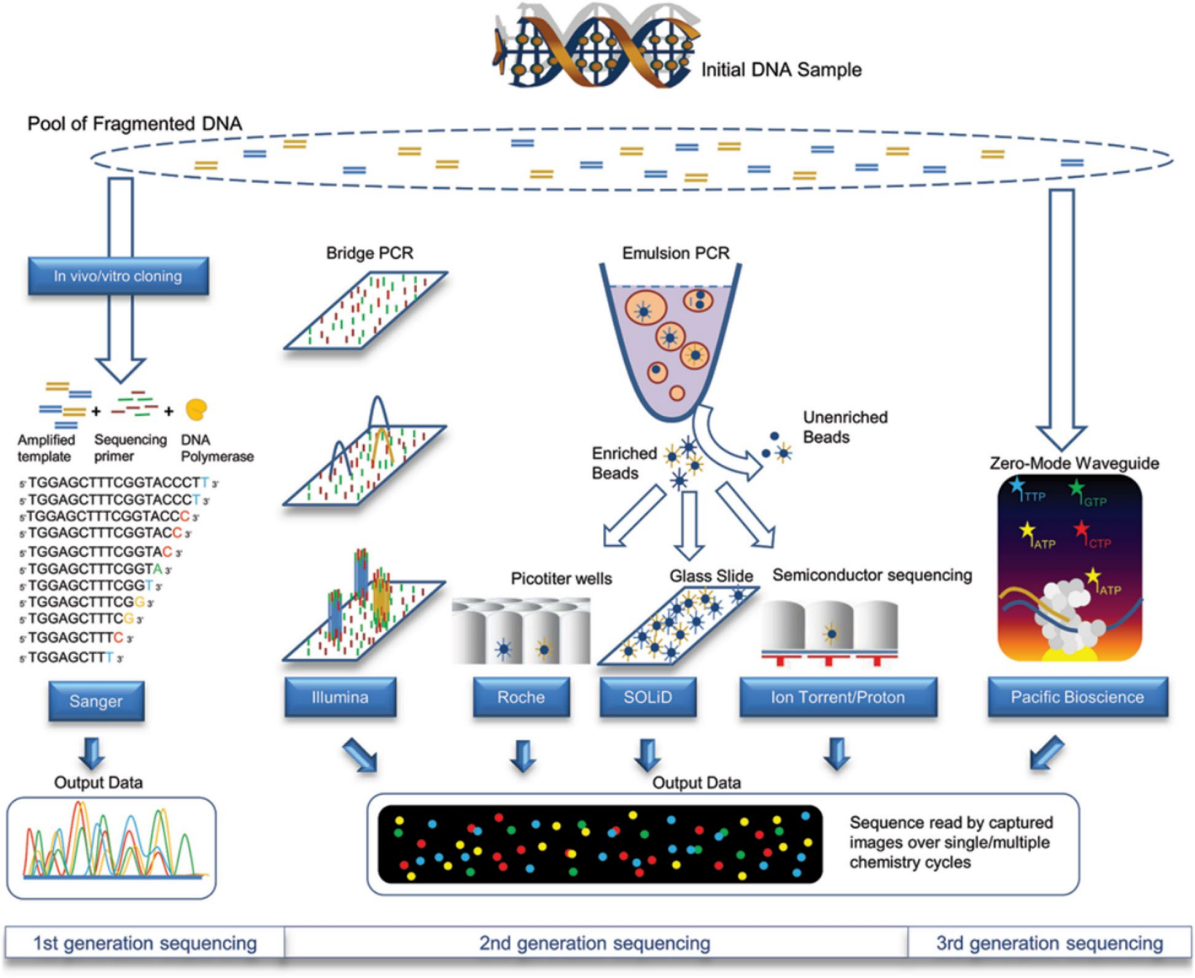
Technical characteristics including sample preparation, sequencing chemistries, and data output formats of different sequencing approaches [60]. The copyright of this image belongs to Reference [60]

Single-cell sequencing analysis technology was developed by the American Anderson Cancer Research Center and Cold Spring Harbor Laboratory in 2011 [4]. Commonly used next-generation sequencing (NGS) platforms include 454 Life Sciences/Roche, Illumina and Applied Biosystems' SOLiD system (Fig. 3) [60]. Single-cell genome sequencing first detects the total amount of amplified products and fragment distribution and constructs a library of qualified samples. Library preparation includes randomly interrupting the amplified products into small DNA fragments, end repair, adding A, adding adapters and PCR amplification to obtain the required library, and sequencing the library concentration and amplified fragment distribution after passing quality inspection.

The basic process of single-cell genome sequencing data analysis is similar to NGS. First, the original offline data is filtered and the quality of the sequencing is evaluated. Then, the filtered data are compared to the reference genome, and the corresponding indicators are quality-controlled. Due to the uneven coverage and high chimerism rate brought about by WGA, the data need to be preprocessed. For example, the nucleic acid library is standardized and can be spliced using traditional splicing methods [60]. At present, data analysis methods developed for single-cell sequencing include Smash Cell, Velvet-SC, and SPAdes. These high-performance computing platforms and bioinformatics methods have overcome the problem of uneven coverage caused by expansion to a certain extent [61–63]. Single-cell genome sequencing can provide information regarding large-scale genomic structure variations, including genome rearrangements, insertions, duplications, inversions and transpositions, as well as genomic structural variation information, such as CNVs and SNVs. SNVs include single base insertions, deletions and mutations. Through these genomic structural variations, tumor driver genes and biomarkers can be found, and the progression of tumorigenesis can also be understood [64].

## Single cell sequencing of circulating tumor cells

As an important indicator of tumor progression, CTC single-cell whole-genome sequencing analysis of the peripheral blood of patients with solid tumors helps to understand the occurrence and development of tumors, especially tumor heterogeneity and drug resistance, and can identify the mechanisms of tumor development. Discovering gene mutations can lead to the discovery of new driver genes, enhance understanding of the clonal origin and evolutionary mechanism of tumors, recognize the genetic sequence differences between tumor subtypes, and contribute to the discovery of new biomarkers [49]. The information obtained through single-cell sequencing is more comprehensive, making up for the deficiencies in tumor stratification based on a single biopsy, and is widely used to assist in the early diagnosis of tumors, the selection of therapeutic drugs, prognosis prediction and relapse monitoring. Tumor diagnosis and prognosis prediction via single-cell sequencing is a noninvasive method (Table 2).

**Table 2:** Summary of single-cell sequencing studies of various cancer CTCs

Tumourtype	Cases	Blood sampling method	CTC enrichment method	CTC			Sequencing method	Analysis Sample			Target gene / sequence	Mutation type	Ref
				CTC single chipCell sorting method	Single			Primary focus	CTC	Metastases / drenchingFawn on			
					Cell Analysis number	WGA method							
Mammary cancer	15+43	CellSave	CellSearch	DEPArray	37+202	Amplil™ WGAKit	qPCR/aCGH/Sanger	√	*√*	X	Erbb2/pik3ca/ allGenomic aCGH analysis	Copy number variationAnomaly / point mutation	[41]
Mammary cancer	2	CellSave	CellSearch	DEPArray	11	Amplil™ WGAKit	Sanger	√	√	X	TP53	point mutation	[7]
Mammary cancer	18	CellSave	CellSearch	DEPArray	115	Amplil™ WGAKit	Sanger	√	*√*	X	PIK3CA	point mutation	[60]
colorectal cancer	6	CellSave	CellSearch	Micromanipulation	37	GenomePlexSingle Cell WGA kit/ GenomiPhiDNAAmplification kit	aCGH/NGS	√	*√*	*(*metastases)	Whole genome aCGHAnalysis of /68 colorectal cancerGuan gene	Copy number variationAnomaly / point mutation	[35]
colorectal cancer	5	CellSave	CellSearch	Micromanipulation	69	GenomePlexSingle Cell WGAkit	Sanger	X	*√*	X	BRAF/KRAS/PIK3CA	point mutation	[6]
Mammary cancerLung cancer	3+2	EDTA	CTC-Chip + doubleCoating of nanomaterialsGel matrix	frequency controlMicroabsorption	NA	NO WGA	Sanger	√	* √*	X	PIK3CA/EGFR	point mutation	[64]
MalignancyPigmented tumor	11	Heparinized blood	Dynabeads	Laser captureMicrodissection	14	NO WGA	Nested PCR+ Sanger	X	√	X	BRAF/KIT	point mutation	[61]
Mammary cancer	17	EDTA	MagSweeper	Under microscopeMicro absorption	185	NO WGA	Nested PCR+ Sanger	* √*	√	*(*metastases)	PIK3CA	point mutation	[65]
Mammary cancer	5	CellSave	CellSearch	DEPArray	≥100	Amplil™ WGAKit	NGS	*√*	*√*	X	ESRI, PIK3CA>TP53 and erbB2 Point mutation; Custom Cancer Hotspot Panel v2 for Amplil WGA DNAfever	point mutation	[66]
pancreatic cancer	12	ACDSolution Atubes	Dynabeads	Nano Velcro/LCM CTCChip	222	REPLI-g SingleCell Kit	Sanger	X	*√*	X	KRAS	point mutation	[67]
Colon cancer	21	EDTA	Oncoquick	DEPArray	NA	Amplil™ WGAKit	Sanger/pyrosequencing	*√*	*√*	X	KRAS	point mutation	[43]
Prostatecancer	1	EDTA	PN-Nano Velcrochip	Laser captureMicrodissection	25	GenomePlexSingle Cell WGAkit	NGS	X	*√*	X	Whole genome exon measurementorder	point mutation	[68]
Prostatecancer	1	Anticoagulated bloodtubes	Red cell lysis + selfSlide adhesion	Micromanipulation	41	GenomePlexSingle Cell WGA kit	NGS	X	*√*	X	whole genome sequencing	Copy number variationdifferent	[63]
ProstateCancer /	2	EDTA	MagSweeper/CelISearch	Micromanipulation	25	MDA	NGS	*√*	√	(lymph node)	Whole genome exon measurementorder	point mutation	[8]
lung cancer	4	CellSave	Cell Search	Under microscopeMicro absorption	24	MALBAC	NGS	*√*	*√*	(metastases)	Whole genome exon measurementorder	Copy number variationHetero / point mutationinsert	[9]
Colon cancer	2	CellSave	CellSearch	Under microscopeMicro absorption	5	MALBAC	NGS	*√*	*√*	*(*lymph node)	whole genome sequencing	Copy number variationHetero / point mutationStructural variation	[62]

### Single-cell sequencing technologies for CTCs

This section describes emerging and important single-cell sequencing technologies for CTCS, such as Hydro-Seq and EPISOT & EPIDROP assays. Yu-Heng Cheng et al. presented Hydro-Seq, a high-efficiency contamination-free cell capture scRNA-seq platform, for the gene expression profiling of CTCs. Hydro-Seq utilizes size-based single-cell capture to prevent bias that may result from molecular CTC selection. This cell capture protocol achieves high cell capture efficiency (72.85 2.64%, representing standard deviation n = 3) for the analysis of a small number of CTCs in a 10 ml blood sample. To enable contamination-free single-cell sequencing, the Hydro-Seq chamber integrates pneumatic valves that allow washing of cell and noncell contaminant clearing chambers on the chip. In addition, the chamber array can be expanded to thousands of chambers for massive parallel analysis. By sequencing 666 CTCs from 21 patients with advanced breast cancer, we validated the utility of Hydro-Seq, identifying cellular heterogeneity as a key biomarker of tumor metastasis and treatment. Hydro-Seq offers the ability to analyze CTCs by single-cell whole transcriptome sequencing for metastasis studies and companion diagnostic applications [65].

Liquid biopsy has been introduced as a new diagnostic concept based on the analysis of CTCsor circulating tumor-derived factors, particularly cell-free tumor DNA (ctDNA). Highly sensitive liquid biopsy assays have since been developed that can be applied to detect and describe minimal residual disease (MRD), which reflects the presence of tumor cells that disseminate from the primary lesion to distant organs in patients lacking any clinical or radiological signs of metastasis, or residual tumor cells remaining after local therapy that ultimately lead to local recurrence. This application is a new frontier in liquid biopsy analysis, which is challenged by the very low concentrations of CTCs in blood samples.

Pantel & Alix-Panabières discussed key techniques such as EPISOT & EPIDROP assays used to detect and characterize CTCs in MRD monitoring and highlighted the current use of CTC analysis to detect and monitor MRD as well as acquire clinical data on therapeutic targets and resistance mechanisms relevant to the management of individual cancer patients [66].

### CTC analysis promotes the accurate typing of tumors

Many previous studies used Sanger sequencing or NGS methods to detect specific gene mutations in CTCs at the single-cell level and found heterogeneity between tumor patients and patients with the same tumor; for example, Sanger sequencing revealed differences between breast cancer CTCs and breast cancer tumor tissue. The level of heterogeneity in PIK3CA gene mutation status is large. In addition, heterogeneous CTC subgroups can also be found based on mutations in the TP53 gene in CTCs [7, 67]. In colorectal cancer studies, BRAF, PIK3CA, and KRAS mutations in different CTCs were found, suggesting the existence of a large level of tumor heterogeneity both between individuals and within the same individual. Similarly, sequencing of BRAF and KIT mutations in malignant melanoma revealed a high degree of heterogeneity between CTCs and tumor tissue [6, 68].

In addition, through genome-wide sequencing and comparative genome hybridization (array comparative genomic hybridization, aCGH) technology, CNV variation patterns in CTCs have been studied at the genome-wide level. Through aCGH analysis of breast cancer CTCs, it was found that the CNV variation in CTCs from different patients with the same pathological type of tumor was highly heterogeneous, suggesting that breast cancer can be more accurately typed according to the CNV variation pattern [46]. A CTC single-cell whole-genome sequencing study on multiple tumors, including gastric cancer, colorectal cancer, breast cancer, and lung cancer, revealed that different CTCs in the same patient had highly consistent genome-wide CNV change patterns, while the CTC CNV variation patterns of different tumors and different pathological types were quite different. Similar to the results obtained in above studies, the CNV change pattern in CTCs of breast cancer patients is complicated, suggesting that CNV analysis can be used as a basis for more accurate typing [69].

### CTC analysis can reveal the mechanism of tumor metastasis

**Fig. 4:**
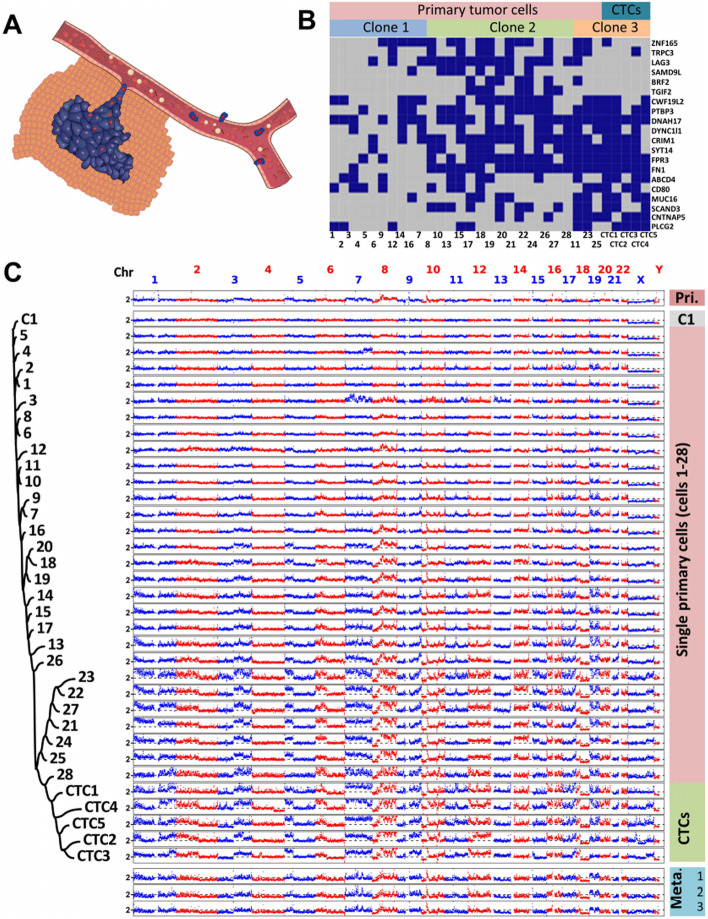
Evolution of SNVs and Large-scale CNAs in Primary Tumour cells and CTCs. **A** Schematic diagram of the manner in which primary tumour cells intravasate and become CTCs. **B** SNVs of primary tumour cells and CTCs. The distribution of 20 non-synonymous mutations was assessed in 28 primary tumour cells (Cells 1–28) and five CTCs (CTCs 1–5) from a colon cancer patient (blue box, mutant; grey box, wild type). Three clones of cells were present according to a probabilistic modelling-based approach. **C** CNA patterns of the primary tumour, one control leukocyte (C1), single primary tumour cells, CTCs, and three lymph node metastases (Pri., primary tumour; Meta., metastases). The copy numbers (blue and red dots) are plotted along the genome at a bin size of 500 kb. The ordinate coordinate represents copy numbers ranging from 0 to 6 (a copy number of more than 6 copies is set to 6). Phylogenetic tree on the left was constructed based on the segmented copy numbers of single cells [70]. The copyright of this image belongs to Reference [70]

Tumor recurrence and metastasis are the main causes of tumor death. CTC single-cell sequencing analysis of CNV and other mutation patterns is helpful to understand the mechanism of tumor metastasis. Heitzer et al. [70] used aCGH to analyze the CNV variation patterns in primary tumors, metastases, and CTCs of colorectal cancer at the single-cell genome level and found that CTCs contained the same CNV variations as the primary tumors and metastases in addition to new CNV variations. Lohr et al. [8] selected 10 patients with metastatic prostate cancer in whom CTCs were not detected in the peripheral blood and performed exome sequencing analysis on tissue samples of the primary tumor and metastatic tumors. Ten mutations, referred to as "early mutations," were found in the primary focus, and 56 mutations, referred to as "metastatic mutations," were found in the metastatic focus. Then, two more patients with metastatic prostate cancer with peripheral blood containing more than 20 CTCs were selected. Exome sequencing analysis was performed on the primary tumors, metastases and CTC single cells, and it was found that 9 of the CTCs were associated with the primary tumors. The same "early mutations" found in the foci and 41 "metastatic mutations" were found in the CTCs, indicating that the CTCs contained both primary tumor and metastasis genetic mutation information; this, it is possible to determine the mechanism of tumor metastasis from the mutations found in CTCs [8]. In 2017, Gao et al. [69] performed genome-wide sequencing analysis on 28 primary tumor cells, 5 CTCs, and 3 metastatic lymph nodes from a colon cancer patient. They found that the CNV variation in 28 tumor cells of the primary focus had greater heterogeneity. That is, the correlation coefficient of the CNV variation of any two primary focus cells was between 0.09 and 0.96; and the CNV variation pattern among the 5 CTCs was similar The CNV variation was close that of three metastatic lymph nodes and is similar to a certain subpopulation of cells in the primary tumor, indicating that the change in the CNV pattern in the process of tumor metastasis is gradually converging, suggesting that there may be only a small group of cells with a higher degree of malignancy Tumor cells can enter the circulatory system from the primary foci and then form metastatic foci. Through comprehensive analysis of SNV, CNV and structural variation, a two-step model of the formation of multi-interval CNVs has been proposed; that is, a multi-interval copy number increase occurs due to a series of replication fork pauses and template transpositions during DNA replication, followed by homologous recombination. Further amplification of this region to a higher copy number reveals the cause of tumor CNV formation at a deeper level (Fig. 4) [70].

### Dynamic monitoring of tumor progression

**Fig. 5:**
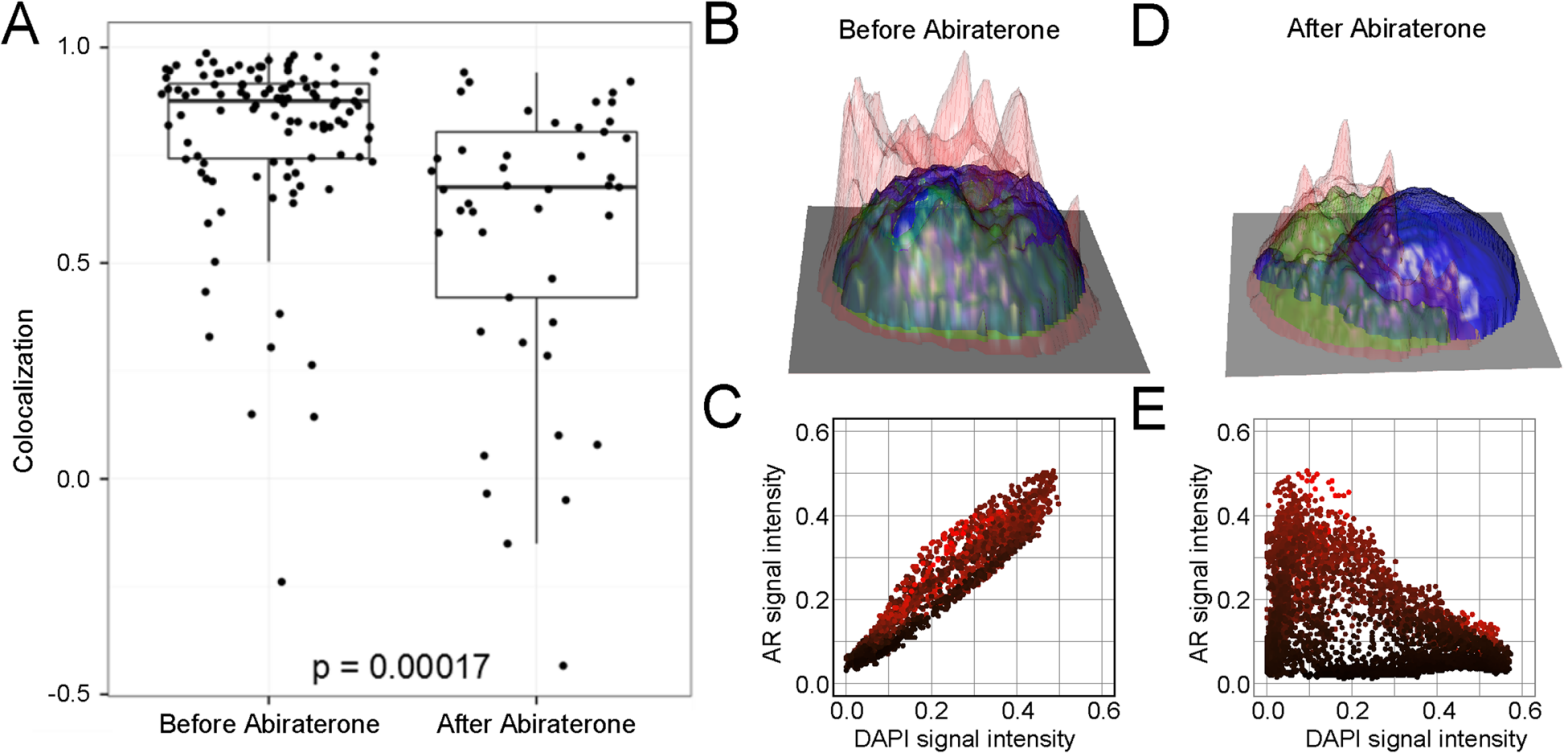
AR subcellular localization changes at the time of disease progression. **A** Comparison of the AR subcellular localization in the CTCs identified in the blood prior to and after nine weeks of abiraterone treatment. Correlation between the AR and DAPI signals within the cell is indicative of AR being colocalized with DAPI, i.e. localized in the cell nucleus. High correlation was generally seen before abiraterone treatment, but a shift to less nuclear stain was observed after nine weeks of treatment (p = 0.00017, Wilcoxon sum-rank test). **B** and **D** Height maps constructed from the pixel intensities of CK (red), AR (green) and DAPI (blue) in representative CTCs to visualize the subcellular localization of AR. The cell in (**B**) was isolated before abiraterone initiation and displays AR staining confined to the nucleus, while cytoplasmic AR staining is observed in the CTC identified at the time of therapeutic relapse (**D**). **C** and **E** Plots of AR versus DAPI signal intensities for each pixel inside the cell in the 406images of the CTCs in (**B**) and (**D**), respectively. Each plot point is colored by the corresponding CK signal intensity. Nuclear localization was observed as positive correlation between the two intensities (**C**), and nuclear exclusion as negative correlation (**E**). All graphs and were done using the ggplot2 and rgl packages in R [73]. The copyright of this image belongs to Reference [73]

As CTCs are an important component of liquid biopsy, an increasing number of studies have tried to use the tumor mutation information from CTCs to guide the clinical treatment of tumors. In prostate cancer, the glucocorticoid receptor (GR) is a prime suspect for acquired therapy resistance, as resistance to the antiandrogen enzalutamide (Enz) can occur through bypass of androgen receptor (AR) blockade by the glucocorticoid receptor (GR)[70]. Prostate cancer (PCa) cells are able to increase GR signaling during anti-androgen therapy and thereby circumvent androgen receptor (AR) blockade and cell death [71, 72]. In 2014, Dago et al. [70] analyzed the whole-genome CNV variation of peripheral blood CTCs in patients with castration-resistant prostate cancer at four treatment time points, before chemotherapy, before treatment with abiraterone, when symptoms were significantly relieved, and when symptoms worsened. Combined with CTC morphology, androgen receptor (androgen receptor, AR) expression levels and other comprehensive analyses, it was found that the change in CNV pattern in CTCs at different periods was significantly different, especially when abiraterone was ineffective and the symptoms were aggravated. CNVs varied greatly, and a subpopulation of CTCs had MYC gene amplifications. The appearance of this subpopulation of malignant CTCs has a significant correlation with the resistance of patients to abiraterone. In contrast to fixed genomic alterations, Shah et al. [71] found that GR-mediated antiandrogen resistance is adaptive and reversible due to regulation of GR expression by a tissue-specific enhancer. GR expression is silenced in prostate cancer by a combination of AR binding and EZH2-mediated repression at the GR locus but is restored in advanced prostate cancers upon reversion of both repressive signals. Puhr et al. [72] identified MAO-A as a directly upregulated mutual epithelial and stromal GR target, which is induced after GC treatment and during PCa progression. Their findings demonstrate that targeting MAO-A represents an innovative therapeutic strategy to synergistically block GR- and AR-dependent PCa cell growth and thereby overcome therapy resistance. Their research showed that CTC single-cell sequencing can be used to dynamically monitor the response of cancer patients to treatment, discover the evolution of tumor cells and disease progression in a timely manner, and establish a new multiparameter comprehensive analysis liquid biopsy program (Fig. 5) [73].

**Fig. 6:**
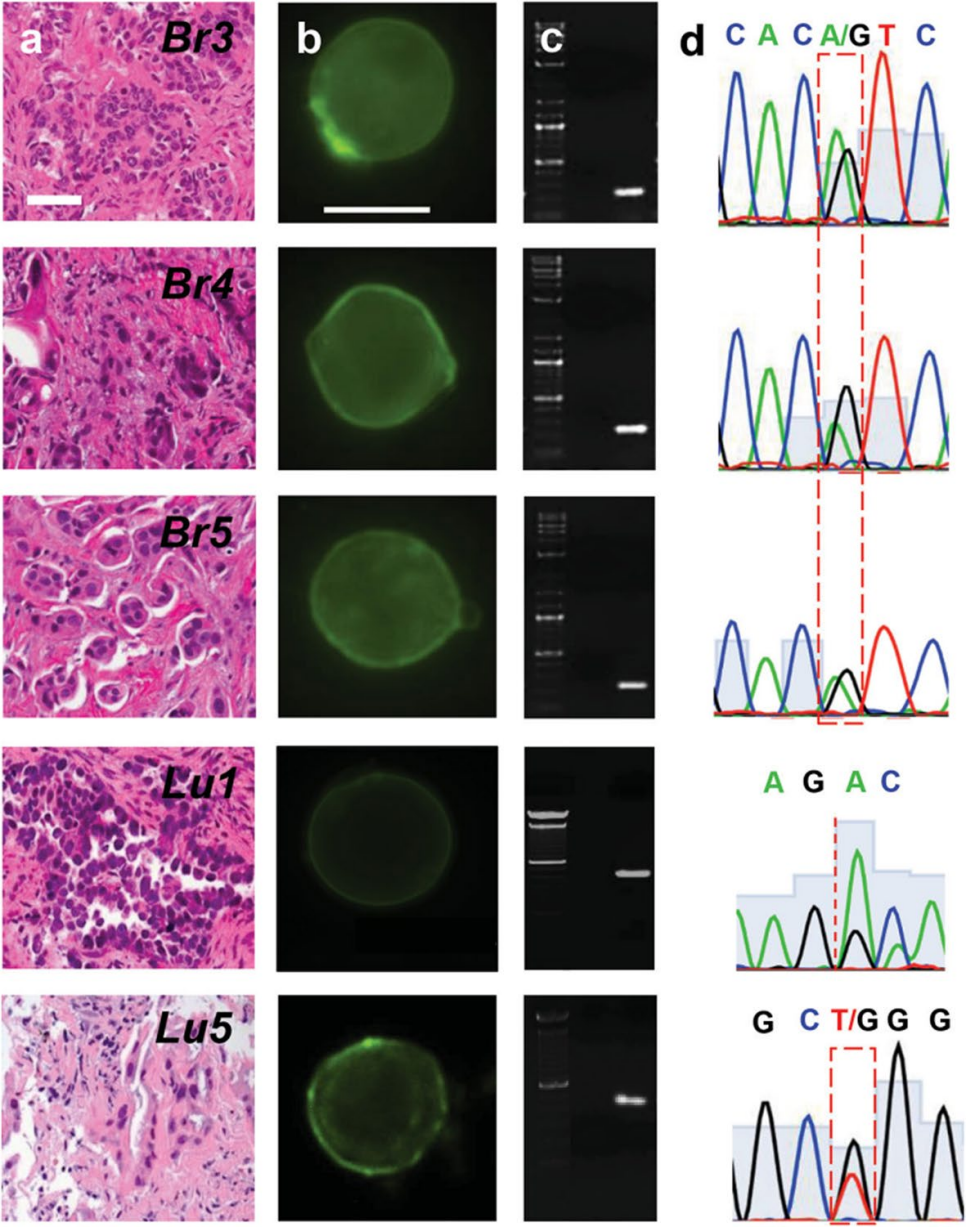
Single cell genomics of CTCs from patients (**a**) H&E staining of the primary tumor of metastatic breast and lung cancer patients. Tissue biopsies were used to determine the presence of DNA mutations on the oncogene PIK3CA and EGFR. **b** Panel of CTCs from the same metastatic breast and lung cancer patients in (**a**). Micrographs of the CTCs identified and subsequently released for molecular analysis using our selective release mechanism (scale bar 10 μm). **c** Micrographs of amplified DNA of the single CTCs shown in (**b**). **d** Sequencing of the amplified DNA from the single CTCs shown in (**b**). The 3140A/G (H1047R) point mutation in the PIK3CA oncogene as well as the exon 19 deletion and the 2573 T/G (L858R) point mutation in the EGFR oncogene were detected at the single cell level [74]. The copyright of this image belongs to Reference [74]

Dynamic monitoring of primary tumor cells, CTCs and tumor metastatic cells through single-cell sequencing can help to elucidate tumor progression in real time in a noninvasive manner, understand the key oncogenes and tumor suppressor genes of tumor patients, and understand the variation in genomic CNVs, as early diagnosis of tumors, dynamic treatment monitoring, and discovery of drug resistance mutations and other important personalized treatment information provide the basis for potential clinical application prospects (Fig. 6) [74–78]. Single-cell sequencing compares the differences between single-cell genomes, transcriptomes, and epigenetic groups in peripheral blood CTCs and tumor primary tumors, metastatic lymph nodes, and metastatic tumors, reducing interference from tumor heterogeneity and increasing understanding of the biology of tumor development. The evolution of this process provides a new perspective [6–9, 68].

### Determining the efficacy of adjuvant therapy

It is also an important application direction of single-cell sequencing analysis to understand the therapeutic effect of tumors through CTC single-cell transcriptome sequencing analysis. In 2014, Ting et al. [77] used CTCs-i Chip to enrich the peripheral blood CTCs of a pancreatic cancer mouse model and analyzed the transcriptome of 75 CTCs at the single-cell level. They comprehensively analyzed the expression levels of marker genes of epithelial cells, hematopoietic cells and endothelial cells, found that there were 7 different cell subpopulations in CTCs, and that the extracellular matrix od mouse pancreatic cancer CTCs had high expression of Dcn, Sparc, Ccdc80, Col1a2, Col3a1 and Timp2 isogenic genes, which are related to the dissemination of the tumor to distant organs. In 2015, this group used the same method to analyze 77 CTCs obtained from the peripheral blood of 13 prostate cancer patients and found that gene expression in CTCs was heterogeneous and included androgen receptor (AR) mutants and splicing-related differential expression of isomers. On this basis, a retrospective analysis of patients using AR inhibitors and their response rate to inhibitors was performed and the results revealed that after using AR inhibitors, patients with CTCs with nonclassical Wnt signaling still showed positive prostate-specific antigens or still needed radiation therapy. The Wnt signaling pathway and its downstream RAC1, RHOA, and CDC42 signals were activated, indicating that the changes in cell signaling pathways in CTCs may be related to the therapeutic response of patients [78]. Compared with CTC single-cell genome sequencing analysis, CTC single-cell transcriptome sequencing analysis is relatively difficult.

## Conclusion and prospects

Single-cell sequencing is a booming emerging technology. In 2013, Science magazine ranked the field of single-cell sequencing among the top six. However, a major challenge in the field is sample size. These cells occur at extremely low frequency, and even after successful enrichment, captured CTCs are of different times (passively and/or actively detached from tumors at different time points) and tumors (primary and/or metastatic tumor), confounding the sequencing results. Furthermore, different single-cell enrichment/library prep/WGA/sequencing technologies used in independent studies serve as another potential source of variation. Single-cell sequencing technology is not yet fully mature; for example, human-generated amplification of whole genome amplification, low coverage, poor reproducibility, allele deletion, false positives and false negatives, as well as errors in sequencing and splicing software occur. Analyzing heterogeneity and clonal evolution, and the discovery of driver genes is challenging. With the continuous optimization of genome-wide amplification methods and the rapid development of bioinformatics methods, these problems will be gradually solved. Amplification methods with higher coverage and better uniformity will promote the development of single-cell genome sequencing technology. For the analysis of single-cell sequencing data, large sample sequencing analysis methods are commonly used, such as Mu Tect, Var Scan, and Monovar. In recent years, researchers have also successfully developed many bioinformatics methods to better analyze high-throughput data. Salehi et al. [79] proposed ddClone based on the analysis results of real and simulated datasets, which analytically integrates NGS and SCS data, leveraging their complementary attributes through a joint statistical inference. Furthermore, technological advances have made it possible to measure spatially resolved gene expression at high throughput. Svensson et al. [80] developed SpatialDE, a statistical test to identify genes with spatial patterns of expression variation from multiplexed imaging or spatial RNA sequencing data, which implemented “automatic expression histology” (the spatial gene clustering approach that enables expression-based tissue histology). Additionally, single-cell transcriptome sequencing and epigenetic sequencing methods are constantly being developed, and single-cell genome sequencing be used in the integrated analysis of single-cell multiomics [81, 82].

Given the key role of CTCs in the development of tumors, with the continuous maturation of single-cell sequencing technology and the standardization of CTC enrichment identification technology, CTC single-cell sequencing analysis will contribute to our understanding of the genetic heterogeneity, evolution, and drug resistance of tumor cells. The integrated analysis of single-cell sequencing combined with other omics provides valuable information and will also promote the development of precision medicine for tumors.

## Data Availability

All datas are available. Please contact us to access if it is needed.
