# Correction to: Using single-cell sequencing technology to detect circulating tumor cells in solid tumors

**DOI:** 10.1186/s12943-022-01564-2

**Published:** 2022-04-18

**Authors:** Jiasheng Xu, Kaili Liao, Xi Yang, Chengfeng Wu, Wei Wu

**Affiliations:** 1 Department of Gastroenterology, Huzhou Central Hospital, Affiliated Huzhou Hospital Zhejiang University School of Medicine, Huzhou, Zhejiang China; 2 Department of Colorectal Surgery and Oncology, Key Laboratory of Cancer Prevention and Intervention, Ministry of Education, The Second Affiliated Hospital, Zhejiang University School of Medicine, Hangzhou, Zhejiang China; 3 Cancer Center, Zhejiang University, Hangzhou Zhejiang, China; 4 Department of Clinical Laboratory, the Second Affiliated Hospital of Nanchang University, No. 1 Minde Road, Nanchang 330006 Jiangxi, China; 5 Department of Oncology, Huzhou Central Hospital, Affiliated Central Hospital Huzhou University, No 1558, Sanhuan North Road, Wuxing District, Huzhou, Zhejiang Province China; 6 Department of Vascular Surgery, the Second Affiliated Hospital of Nanchang University, No. 1 Minde Road, Nanchang 330006 Jiangxi, China; 7 Department of Gastroenterology, Huzhou Central Hospital, Affiliated Central Hospital Huzhou University, No 1558, Sanhuan North Road, Wuxing District, Huzhou, Zhejiang Province China

**Correction to: Mol Cancer 20, 104 (2021)**

**https://doi.org/10.1186/s12943-021-01392-w**

Following publication of the original article [1], the authors requested that the following adjustments be made:

That Shuwen Han be removed from the author list; Wei Wu is now listed as the corresponding author.

That the Acknowledgements section be updated to include the following wording: "Thanks to Han Shuwen for his writing guidance and paper revision."

The corrected author list can be found above. The correction does not have any effect on the final conclusions of the paper. The original article has been corrected.

## References

[1] Xu J, Liao K, Yang X (2021). Using single-cell sequencing technology to detect circulating tumor cells in solid tumors. Mol Cancer.

